# Bimodal Extended Kalman Filter-Based Pedestrian Trajectory Prediction

**DOI:** 10.3390/s22218231

**Published:** 2022-10-27

**Authors:** Chien-Yu Lin, Lih-Jen Kau, Ching-Yao Chan

**Affiliations:** 1 Department of Electronic Engineering, National Taipei University of Technology, Taipei 106344, Taiwan; 2 California Partners for Advanced Transportation Technology, University of California, Berkeley, CA 94804, USA

**Keywords:** pedestrian trajectory prediction, bimodal extended Kalman filter, point-cloud

## Abstract

We propose a pedestrian trajectory prediction algorithm based on the bimodal extended Kalman filter. With this filter, we are able to make full use of the dual-state nature of the pedestrian movement, i.e., the pedestrian is either moving or remains stationary. We apply the dual-mode probability model to describe the state of the pedestrian. Based on this model, we construct the proposed bimodal extended Kalman filter to estimate pedestrian state distribution. The filter obtains the state distribution for each pedestrian in the scene, respectively, and use that state distribution to predict the future trajectories of all the people in the scene. This prediction method estimates the prior probability of each parameter of the model through the dataset and updates the individual posterior probability of the pedestrian state through the bimodal extended Kalman filter. Our model can predict the trajectory of every individual, by taking the social interaction of pedestrians as well as the surrounding physical obstacles into account, with less than fifty model parameters being used, while with the limited parameter, our model could be nearly accurate as other deep learning models and still be comprehensible for model users.

## 1. Introduction

Pedestrian trajectory prediction and pedestrian state filtering are often applied in various fields such as self-driving systems [1,2] and pedestrian tracking in surveillance systems [3,4]. In the self-driving system, the state estimation and the tracking algorithm play an important role in the system.

A good prediction algorithm can help the autonomous driving system compute a better plan according to the encountered situation [2]. By performing tracking for the moving pedestrian around its surroundings, and taking the prediction of the future trajectories of pedestrians into account, the path planner would able to avoid the potential collision with the moving pedestrians or planning in a less oppressive route to bypass its surrounding pedestrians.

On the other hand, the prediction algorithm also plays a key component in the Multi-Object Tracking (MOT) algorithm in surveillance systems. A good prediction model would lower the trajectory prediction error for trackers [5] and therefore help the data association and tracker to track the moving object more accurately in the Multi-Object Tracking algorithm in the surveillance system.

Recently, deep learning-based models such as social GAN [6] showed their ability to predict socially acceptable trajectories via the deep neural network. These models often suffer from lacking the interpretability of the prediction models. It is challenging for the model users to comprehend the models, as the meaning of the weights and biases in the deep neural networks are not obvious. Such a situation motivates us to pursue a model with interpretability while keeping the multi-modality and the social behavior of pedestrians in mind.

### Literature Review

For the current pedestrian trajectory prediction algorithms, the algorithms could be divided into two categories: one is the deep learning-based algorithm [6,7,8,9,10] and the other is the traditional model-based algorithm [11,12,13,14].

Deep learning-based pedestrian prediction models [6,7,8,9,10] are built on top of various neural network structures. This kind of algorithm works by reading the pedestrian trajectories and the context of the surrounding environment, generating the features via deep neural networks, and finally predicting the future pedestrian trajectories based on these features. On the other hand, the traditional algorithms [11,12,13,14] predict the trajectories by first manually analyzing the context in which the pedestrians are moving. After that, the prediction model is built to extract the features based on the analysis and predict the future trajectories on top of the extracted features.

For the deep learning-based pedestrian trajectory prediction models, take the social LSTM [7] as an example. The social LSTM [7] is a deep learning-based pedestrian trajectory prediction model and is proposed by Alahi Alexandre  et al. They use the LSTM [15] to consume the observed trajectories and construct the trajectory features for every individual in the scene, and propose a social pooling mechanism to calculate the features of the interactions between pedestrians. This algorithm is a classic deep learning-based method that takes social interaction into consideration.

Continuing from the social LSTM [7], Agrim Gupta et al. propose the social GAN [6]. They employ the GAN [16] architecture in solving the pedestrian trajectory prediction problem. They also adjust the social pooling mechanism from that of social LSTM [7]. A new maximum pooling-based social pooling mechanism is proposed to construct social features. Furthermore, they improve the pooling mechanism and reduce the time of social feature calculation by computing interaction features per scene instead of computing the interaction for every frame.

For the traditional pedestrian algorithms, Dirk Helbing and Peter Molnar propose the concept of social force and use this concept to build the social force model. They treat pedestrians as moving particles, which are affected by the social force from the surrounding. For example, a pedestrian is attracted by the goal and therefore he/she is affected by the attraction force from the goal and trying to move toward the goal. Furthermore, a pedestrian might want to avoid collision with obstacles and other pedestrians, which is modeled as the repulsive force to the pedestrians. The repulsive force discourages the pedestrian from moving in that direction. The social force model is built with the consideration of these forces in its model dynamics.

Yamaguchi Kota et al. propose an energy-based pedestrian behavior model [14]. They construct a series of energy functions to describe how pedestrians see the environment and use the energy function to model the motion of the pedestrians. For example, pedestrians prefer moving at a certain speed and maintaining their current velocity, pedestrians want to move toward the destination, and pedestrians in the same group tend to maintain the same speed and same moving direction. They assume that pedestrians have the nature to move in a way such that the energy is minimized. Therefore they propose to take the negative gradient of the mixture energy function as the pedestrian’s velocity and use the velocity to predict future trajectories.

As the traditional model-based pedestrian trajectories prediction models have the advantage of comprehensibility for the model users. The model users can see how the model work. Furthermore, it is much easy for the users to tweak the internal parameters compared to that of the deep learning-based models. Therefore, this study chooses the traditional model as our research direction.

In this research, we employ the concept of the social force model [11] and propose the use of a dual-mode Kalman filter. We consider the static and moving mode of pedestrian motion and predict the trajectory according to the mode of the pedestrians.

## 2. Materials and Methods

### 2.1. Dataset Preparation

In this research, we aim to create a motion model of pedestrians and predict their future trajectories based on observed data in the scene. In order to construct the pedestrian trajectory prediction algorithm, optimize the model parameters, or evaluate the performance of the algorithm, relevant data need to be prepared for follow-up research work. There are two parts of the data to be prepared in this research: one is the pedestrian trajectories in the scene, and the other one is the map of static obstacles in the environment.

**Figure 1:**
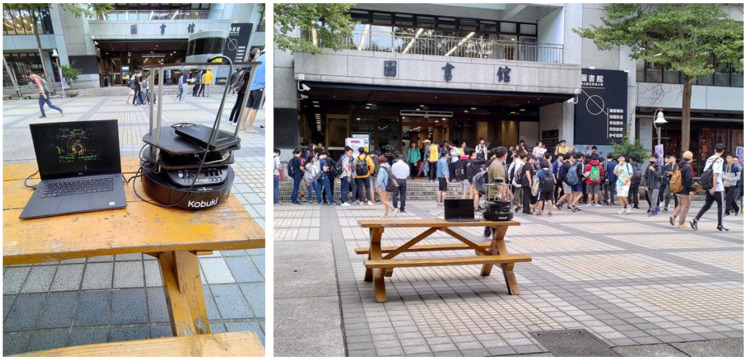
The setup of the Velodye LiDAR sensor.

To build the dataset for the experiment, we first collect the data at the front square of the National Taipei University of Technology (NTUT), with the help of the Velodyne VLP-16 3D LiDAR sensor. We installed the LiDAR on top of a table, which is near the center of the square, so that the sensor would have a better field of view of the surrounding pedestrians. The environment and setup is shown in Figure 1. The point-cloud data is then collected at the frame rate of 10 Hz for 90 min starting at 16:00 on 1 November 2020.

After the point-cloud data is collected, the data have to be processed into the format that is suitable for our research needs. The data would be processed and transformed into an environment obstacle map and pedestrian trajectories dataset. To extract the map and trajectories, a list of steps is executed sequentially.

To extract the trajectories data, firstly, the background point-cloud extraction is performed on all the collected frames of point-clouds by building the histogram of ray-hitting distance for every direction of the laser ray and extracting the most frequently hitting point for every ray direction. In such a way, we are able to extract the background point cloud.

**Figure 2:**
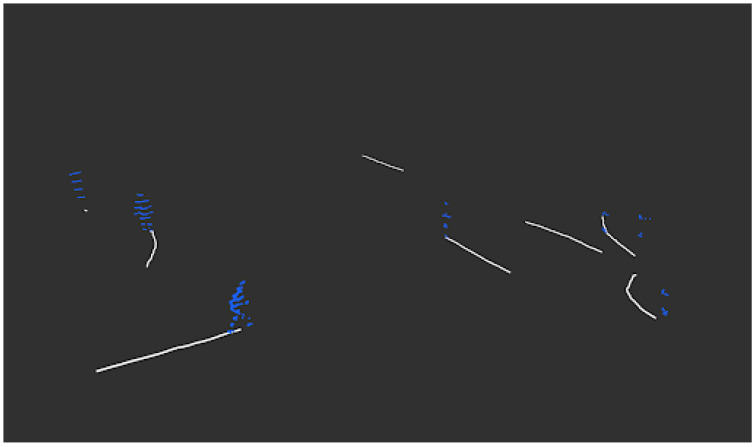
Example of tracked pedestrian trajectories.

Secondly, after the background point-cloud is retrieved, we take this background and the collected point-clouds as input. We apply the background subtraction method to extract the foreground pedestrian point-clouds. Then we perform the clustering and multi-object tracking algorithm to extract the pedestrian trajectories as shown in the algorithm [17]. The tracked cluster and trajectories are shown in Figure 2.

Finally, the extracted trajectories are manually adjusted and annotated, so that the trajectories would not suffer from the errors during the tracking algorithm in the previous step. The frame rate for the trajectories data is also down-sampled to 2.5 frames per second, which is aligned with that in trajectories dataset [18,19].

**Figure 3:**
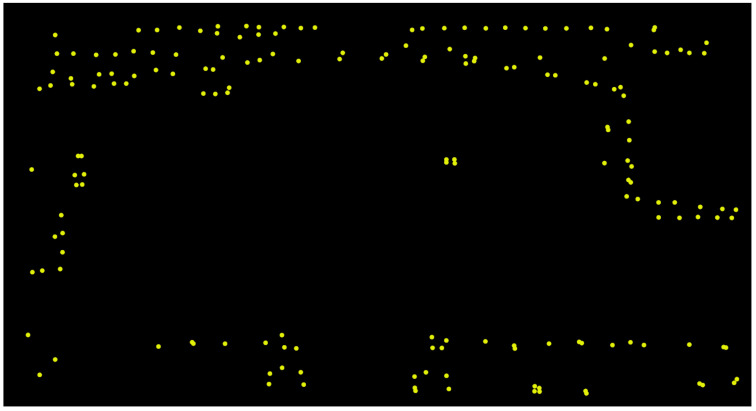
Down-sampled obstacle map.

To obtain the environment obstacle map, we perform the SLAM algorithm [1] to build the point-cloud map of the experiment and align the point-cloud map to the background map with the scan-matching algorithms [20,21]. Then, we keep the points in our range of interests and flatten the 3D point cloud into a 2D one. Finally, we down-sample the point cloud to reduce the points and obtain the obstacle map eventually. This map represents the static obstacles in the experiment environment as shown in Figure 3.

### 2.2. Proposed Model

We proposed a bimodal extended Kalman filter-based pedestrian trajectory prediction model. This model adopts the bimodal extended Kalman filter, which is a special implementation of the Bayes filter. This filter is applied to encode the observed trajectories of the pedestrians and their surrounding obstacles into the state representation. After the state representation is encoded via the filter, the representation is then passed to the decoder to predict the future trajectories of the pedestrians.

#### 2.2.1. Problem Formulation

In this study, we proposed a pedestrian trajectories prediction model G. The model consumes the observed pedestrian trajectories *X* and obstacles *O* of their walking environment, and predict the future trajectories $\hat{Y}$ of the pedestrians in the scenes. We formulate the problem as follows:

(1) $$\hat{Y}=G\left(X,O\right)$$

1. Observed trajectories $X≜{\langle \zeta_{\mathrm{obs},i}\rangle }_{i=1:I}$: The observed trajectories *X* are the collection of the observed pedestrian trajectory $\zeta_{\mathrm{obs},i}$. The pedestrian index number *i* is used in the symbol subscript to denote a specific pedestrian trajectory in the scene. The pedestrian trajectory $\zeta_{\mathrm{obs},i}≜{\langle p_{i}^{t}\rangle }_{t=1:t_{\mathrm{obs}}}$ is defined as a sequence of 2D positions $p_{i}^{t}≜\langle x_{i}^{t},y_{i}^{t}\rangle \in R^{2}$. It describes how a specific pedestrian is being located throughout a specific period of frames, which starts from frame t=1 to $t=t_{\mathrm{obs}}$. In this paper, we set the observed sequence length $t_{\mathrm{obs}}$ to 8.
2. Predicted trajectories $\hat{Y}≜{\langle {\hat{\zeta }}_{\mathrm{pred},i}\rangle }_{i=1:I}$: The predicted trajectories $\hat{Y}$ are the collection of the predicted pedestrian trajectory ${\hat{\zeta }}_{\mathrm{pred},i}$, which are generated via the prediction model G. The pedestrian index number *i* is used in the symbol subscript to denote a specific pedestrian trajectory in the scene. The pedestrian trajectory ${\hat{\zeta }}_{\mathrm{pred},i}≜{\langle {\hat{p}}_{i}^{t}\rangle }_{t=t_{\mathrm{obs}}+1:t_{\mathrm{obs}}+t_{\mathrm{pred}}}$ is defined as a sequence of 2D positions ${\hat{p}}_{i}^{t}≜\langle x_{i}^{t},y_{i}^{t}\rangle \in R^{2}$. It describe how a specific pedestrian is located throughout a specific period of time frames, which starts from frame $t=t_{\mathrm{obs}}$ to $t=t_{\mathrm{obs}+\mathrm{pred}}$. In this paper, we set the predicted sequence length $t_{\mathrm{pred}}$ to 8.
3. Ground-truth predicted trajectories $Y≜{\langle \zeta_{\mathrm{pred},i}\rangle }_{i=1:I}$: The ground-truth predicted trajectories represent the pedestrian trajectories in the future like the predicted trajectories, but they are not generated by a prediction model G. Instead, they describe the pedestrians’ real trajectories in the dataset throughout the same time frames as the predicted trajectories.
4. Environment Obstacles $O≜{\langle o_{k}\rangle }_{k=1:K}$: The environment obstacles are a set of obstacles in the pedestrian walking environment. An obstacle is a 2D point representing where the obstacle is located.

#### 2.2.2. State Representation

In our work, we choose the setting for the state so that it is able to represent the state information of that pedestrian in the specific frame. The state s≜〈m,x〉 is a tuple that consists of two variables.

The first variable m∈{0,1}, named mode, is the discrete random variable describing the pedestrian’s moving mode. We consider the pedestrians has two moving modes, hence this variable is a binary variable. We define m=0 being the static mode, any pedestrian in this mode prefers to stay in the same location. On the other hand, we defined the mode m=1 as the moving mode, any pedestrian in such mode would prefer moving but not staying in the same location.

The second variable $x≜{\left[p^{T}\;v^{T}\right]}^{T}\in R^{4}$, named base state, is a 4-dimensional vector which contains the 2-dimensional position p and 2-dimensional velocity v of the pedestrian.

With such a setting, we are able to estimate the state and predict the trajectories in a mode-aware manner.

#### 2.2.3. State Distribution

To represent the belief distribution of the state variable, we first decompose the belief state distribution bel(s) into the product of the mode distribution p(m) and the base state distribution p(x∣m) conditioning on the mode. For the mode distribution p(m), it is a Bernoulli distribution; therefore, the distribution is parameterized with the weight parameter $w_{m}$ to represent the probability under the specific mode. For the mode-conditioned base state distribution p(x∣m), we parameterize the distribution as a multivariate normal distribution, with $\mu_{m}$ as its mean and $\Sigma_{m}$ as its covariance matrix. The subscript *m* shows the mode that the parameter is conditioning on. We denote the belief state distribution as $bel\left(s\right)=N\left(x;\mu_{m},\Sigma_{m}\right)w_{m}$.

#### 2.2.4. The Proposed Model

For the proposed prediction model, we first encode the trajectories into the belief state distribution, which is the state distribution built on top of the observed data. To calculate the state distributions for the pedestrians in the scene. We introduce the bimodal extended Kalman filter to help encode the observed trajectories into the state distributions. The algorithm is performed as follows.

Just like the extended Kalman filter [22] is an implementation of the Bayes filter, the proposed bimodal extended Kalman filter is an implementation of the bimodal Bayes filter. We will first show the algorithm of the bimodal Bayes filter, as it provides the overview of the workflow for the bimodal extended Kalman filter. Then we will illustrate the algorithm of the bimodal extended Kalman filter.

The bimodal Bayes filter is a specialization of the Bayes filter in which the state consists of a binary mode variable and a continuous multi-dimensional variable. The estimated state is processed in two phases. For the first phase, the current state distribution is predicted based on the previous state distribution with the help of the state prediction model. In the case of the bimodal extended Kalman filter, there are three minor steps in the prediction phase. Firstly, the mode is being predicted with the mode transition model $p\left(m^{\prime }|m\right)≜t_{m^{\prime }|m}$. Secondly, the base state for each mode is predicted via the mode-specific motion model $x^{\prime }=g_{m^{\prime }|m}\left(x,\epsilon \right)$. Finally, the predicted base states would be merged into the predicted mode-conditioned base state distribution. After the first phase is completed, the second phase is followed. The second phase is the correction phase, the predicted distribution would be corrected based on the observed data in this. In the correction phase, there are also three minor steps. Firstly, the likelihood function of the state is being computed based on the observed pedestrian position z and the observation model $z=h_{m^{\prime }}\left(x,\delta \right)$. Then, the mode is corrected with the likelihood function via the Bayes rule. Finally, the predicted base state is corrected.

To estimate the state with the bimodal Bayes filter, one should provide the previous belief state distribution and the state observation, with a properly configured state transition model and observation model. The state-transition model takes the previous state as input and computes the current state. The state transition model contains two parts: one is the mode transition model and the other one is the base state transition model. The mode transition model $p(m^{\prime }|m)$ represent the mode transition probability and is parameterized as $t_{m^{\prime }|m}$. The base state transition model $g_{m^{\prime }|m}$ is a function that takes the previous base state x and a standard normal noise vector ϵ as input and produces the current base state $x^{\prime }$, and shows the base state transition from the previous mode *m* to the current $m^{\prime }$. The transition process could be denoted as $x^{\prime }∼p\left(x^{\prime }|x,m,m^{\prime }\right)\Leftrightarrow x^{\prime }=g_{m^{\prime }|m}\left(x,\epsilon \right),\epsilon ∼N\left(0,I\right)$. The observation model models the observation process of the state. It takes the state *s* and the observation noise vector δ as input, and produces the observation vector z as output. The observation process could be denoted as $p\left(z^{\prime }|x^{\prime },m^{\prime }\right)\Leftrightarrow z^{\prime }=h_{m^{\prime }}\left(x^{\prime },\delta \right),\delta ∼N\left(0,I\right)$. The pseudo-code for this algorithm is shown in Algorithm 1.

As the bimodal extended Kalman filter is the implementation of the bimodal Bayes filter, the bimodal extended Kalman filter further assumes the base state is normally distributed and linearizes the base state transition model as well as the observation model to approximate posterior distribution, which is similar to how the extended Kalman filter [22] extends the Kalman filter [23]. The prediction phase and the correction phase are performed sequentially in the filtering algorithm. The pseudo-code for this algorithm is shown in Algorithm 2.
**Algorithm 1:** Bimodal Bayes Filter.  **Input**: belief distribution bel(s) of the previous state *s*  **Output**: belief distribution $bel(s^{\prime })$ of the current state $s^{\prime }$  // **Prediction step**
  $\bar{bel}\left(s^{\prime }\right)=\bar{bel}\left(x^{\prime }|m^{\prime }\right)\bar{bel}\left(m^{\prime }\right)$ where     // **Mode Prediction**    $\bar{bel}\left(m^{\prime }\right)=\sum_{m}p\left(m^{\prime }|m\right)bel\left(m\right)$    // **Base State Prediction**    $p\left(x^{\prime }|m,m^{\prime }\right)=\int p\left(x^{\prime }|x,m,m^{\prime }\right)bel\left(x|m\right)dx$    $p\left(m|m^{\prime }\right)=\frac{p\left(m^{\prime }|m\right)bel\left(m\right)}{\bar{bel}\left(m^{\prime }\right)}$    $\bar{bel}\left(x^{\prime }|m^{\prime }\right)=\sum_{m}p\left(x|m,m^{\prime }\right)p\left(m|m^{\prime }\right)$  // **Correction step**  $bel\left(s^{\prime }\right)=bel\left(x^{\prime }|m^{\prime }\right)bel\left(m^{\prime }\right)$ where    // **Mode Correction**    $p\left(z^{\prime }|m^{\prime }\right)=\int p\left(z^{\prime }|x^{\prime },m^{\prime }\right)\bar{bel}\left(x^{\prime }|m^{\prime }\right)dx^{\prime }$    $\eta_{m^{\prime }}=1/\sum_{m^{\prime }}p\left(z^{\prime }|m^{\prime }\right)\bar{bel}\left(m^{\prime }\right)$    $bel\left(m^{\prime }\right)=\eta_{m^{\prime }}p\left(z^{\prime }|m^{\prime }\right)\bar{bel}\left(m^{\prime }\right)$    // **Base State Correction**    $\bar{bel}\left(z^{\prime },x^{\prime }|m^{\prime }\right)=p\left(z^{\prime }|x^{\prime },m^{\prime }\right)\bar{bel}\left(x^{\prime }|m^{\prime }\right)$    $bel\left(x^{\prime }|m^{\prime }\right)=\bar{bel}\left(z^{\prime },x^{\prime }|m^{\prime }\right)/p\left(z^{\prime }|m^{\prime }\right)$  **return** 
$bel(m^{\prime },x^{\prime })$

To estimate the current state distribution of the algorithm, the belief distribution of the previous state $bel\left(s\right)=N\left(x;\mu_{m},\Sigma_{m}\right)w_{m}$ is passed into the filter as the input, and it is followed by the prediction phase (Algorithm 2 line 1) and the correction phase (Algorithm 2 line 10), and finally the belief distribution of the current state $bel\left(s^{\prime }\right)=N\left(x^{\prime };\mu_{m^{\prime }}^{\prime },\Sigma_{m^{\prime }}^{\prime }\right)w_{m^{\prime }}^{\prime }$ is produced as the output.

In the prediction phase, the mode is predicted using the Bayes rule with the mode transition model $p\left(m^{\prime }|m\right)≜t_{m^{\prime }|m}$ (Algorithm 2 lines 2 and 3). Then, the base state distribution $p\left(x^{\prime }|m,m^{\prime }\right)≜N\left(x^{\prime };{\bar{\mu }}_{m^{\prime },m}^{\prime },{\bar{\Sigma }}_{m^{\prime },m}^{\prime }\right)$ is predicted with the linearized base state transition model (Algorithm 2 lines 4–6). The Jacobian matrices *G* and *E* of the base state transition model $g_{m^{\prime },m}$ with respect to the base state x and the standard multivariate normal noise ϵ are computed. These Jacobian matrices are used for computing the first-order approximation of the predicted state distribution. The predicted modal mean ${\bar{\mu }}_{m^{\prime },m}^{\prime }$ is approximated by the application of the base state transition model $g_{m^{\prime },m}$ at the mean of previous base state $\mu_{m}$ and the zero mean of the standard multivariate-normal noise. The predicted modal covariance ${\bar{\Sigma }}_{m^{\prime },m}^{\prime }$ is computed as the sum of the transformed previous base state covariance $G\Sigma_{m}G^{T}$ and the transformed noise covariance $EE^{T}$. Finally, the predicted modal base state distributions are merged into the predicted base state distribution. As the mixture of two normal distributions may not distribute normally, the mixture normal distribution is merged approximately by preserving the mean and covariance of the mixture distribution. After the prediction phase is completed, the correction phase is followed (Algorithm 2 lines 8 and 9).
**Algorithm 2:** Bimodal Extended Kalman Filter.  **Input**: belief distribution $bel\left(s\right)=N\left(x;\mu_{m},\Sigma_{m}\right)w_{m}$ of the previous state *s*  **Output**: belief distribution $bel\left(s^{\prime }\right)=N\left(x^{\prime };\mu_{m^{\prime }}^{\prime },\Sigma_{m^{\prime }}^{\prime }\right)w_{m^{\prime }}^{\prime }$ of the current state $s^{\prime }$  // **Prediction step**  $\bar{bel}\left(s^{\prime }\right)=N\left(x^{\prime };{\bar{\mu }}_{m^{\prime }}^{\prime },{\bar{\Sigma }}_{m^{\prime }}^{\prime }\right){\bar{w}}_{m^{\prime }}^{\prime }$ where     // **Mode Prediction**    ${\bar{w}}_{m^{\prime }}^{\prime }=\sum_{m}t_{m^{\prime }|m}w_{m}$    ${\bar{w}}_{m^{\prime },m}^{\prime }=\frac{t_{m^{\prime }|m}w_{m}}{{\bar{w}}_{m^{\prime }}^{\prime }}$    // **Predict the base state for each mode**    $G=G_{m^{\prime },m}=\frac{\partial g_{m^{\prime },m}}{\partial x}$    $E=E_{m^{\prime },m}=\frac{\partial g_{m^{\prime },m}}{\partial \epsilon }$    ${\bar{\mu }}_{m^{\prime },m}^{\prime }=g_{m^{\prime },m}\left(\mu_{m},0\right)$    ${\bar{\Sigma }}_{m^{\prime },m}^{\prime }=G\Sigma_{m}G^{T}+EE^{T}$    // **Merge the base state**    ${\bar{\mu }}_{m^{\prime }}^{\prime }=\sum_{m}w_{m^{\prime },m}\mu_{m^{\prime },m}$    ${\bar{\Sigma }}_{m^{\prime }}^{\prime }=\sum_{m}{\bar{w}}_{m^{\prime },m}^{\prime }{\bar{\Sigma }}_{m^{\prime },m}^{\prime }+\sum_{m}{\bar{w}}_{m^{\prime },m}^{\prime }{\bar{\mu }}_{m^{\prime },m}^{\prime }{\bar{\mu }}_{m^{\prime },m}^{\prime T}-{\bar{\mu }}_{m^{\prime }}^{\prime }{\bar{\mu }}_{m^{\prime }}^{\prime T}$  // **Correction step**  $bel\left(s^{\prime }\right)=N\left(x^{\prime };\mu_{m^{\prime }}^{\prime },\Sigma_{m^{\prime }}^{\prime }\right)w_{m^{\prime }}^{\prime }$ where     // **Compute observation distribution**  $p\left(z^{\prime }|m^{\prime }\right)≜N\left(z^{\prime };{\bar{\nu }}_{m^{\prime }}^{\prime };{\bar{\Pi }}_{m^{\prime }}^{\prime }\right)$    $H=H_{m^{\prime }}=\frac{\partial h_{m^{\prime }}}{\partial x}$    $D=D_{m^{\prime }}=\frac{\partial h_{m^{\prime }}}{\partial \delta }$    ${\bar{\nu }}_{m^{\prime }}^{\prime }=h_{m^{\prime }}\left({\bar{\mu }}_{m^{\prime }}^{\prime },0\right)$    ${\bar{\Pi }}_{m^{\prime }}^{\prime }=H{\bar{\Sigma }}_{m^{\prime }}^{\prime }H^{T}+DD^{T}$    // **Mode correction**    $\eta =1/\sum_{m^{\prime }}{\bar{w}}_{m^{\prime }}^{\prime }N\left(z^{\prime };{\bar{\nu }}_{m^{\prime }}^{\prime },{\bar{\Pi }}_{m^{\prime }}^{\prime }\right)$    $w_{m^{\prime }}^{\prime }=\eta \cdot {\bar{w}}_{m^{\prime }}^{\prime }N\left(z^{\prime };{\bar{\nu }}_{m^{\prime }}^{\prime },{\bar{\Pi }}_{m^{\prime }}^{\prime }\right)$    // **Base state correction**    $K={\bar{\Sigma }}_{m^{\prime }}^{\prime }H^{T}{\bar{\Pi }}_{m^{\prime }}^{\prime -1}$    $\mu_{m^{\prime }}^{\prime }={\bar{\mu }}_{m^{\prime }}^{\prime }+K\left(z^{\prime }-{\bar{\nu }}_{m^{\prime }}^{-}\right)$    $\Sigma_{m^{\prime }}^{\prime }=\left(I-KH\right){\bar{\Sigma }}_{m^{\prime }}^{\prime }$  **return**
$w_{m^{\prime }}^{\prime },\mu_{m^{\prime }}^{\prime },\Sigma_{m^{\prime }}^{\prime }$

In the correction phase, the predicted distribution would be corrected based on the observed data $z^{\prime }$. There are also three steps in this phase. First, the observation distribution $p\left(z^{\prime }|m^{\prime }\right)≜N\left(z^{\prime };{\bar{\nu }}_{m^{\prime }}^{\prime };{\bar{\Pi }}_{m^{\prime }}^{\prime }\right)$ of the state is computed based on the observed data $z^{\prime }$ and the linearized observation model $h_{m^{\prime }}$ (Algorithm 2 lines 11–14). The Jacobin matrices *D* and *H* of the observation $h_{m^{\prime }}$ with respect to the base state $x^{\prime }$ and the standard multivariate normal noise δ are computed. The mean ${\bar{\mu }}_{m^{\prime },m}^{\prime }$ is approximated by the application of the observation model $h_{m^{\prime }}$ at the mean of the predicted base state ${\bar{\mu }}_{m^{\prime }}^{\prime }$ and the zero mean of the standard multivariate-normal noise. The covariance ${\bar{\Pi }}_{m^{\prime }}^{\prime }$ is computed as the sum of the transformed predicted base state covariance $H{\bar{\Sigma }}_{m^{\prime }}^{\prime }H^{T}$ and the transformed noise covariance $DD^{T}$. Then, the mode is corrected via the Bayes rule (Algorithm 2 lines 15–16). Finally, the Kalman gain *K* is computed and the predicted base state distribution is corrected with the Kalman gain (Algorithm 2 lines 17–19).

In our setting, the base state x represents the pedestrian’s position p and velocity v, the mode *m* to represent the static and the moving mode pedestrian, and the observation z represents the position of the pedestrian. The required models for the bimodal extended Kalman filter are specified as follows:

1. For the observation model, it is parameterized with the standard deviation $\sigma_{p}$ of the observation noise.
  (2) $$\begin{array}{cc} z^{\prime } & =h_{m^{\prime }}\left(x^{\prime },\delta \right)≜p^{\prime }+\sigma_{p}\delta ,\;\mathrm{where}\;\delta ∼N\left(0,I\right) \end{array}$$
2. For the mode transition model, it is defined as:
  (3) $$p\left(m^{\prime }|m\right)≜t_{m^{\prime }|m}$$
3. For the base state transition model, we apply different motion-model according to the mode. When current mode $m^{\prime }$ is in static mode, we model the motion as constant position model. On the other hand, when current mode $m^{\prime }$ is in moving mode, we apply the social force model [11] as the motion model. For both scenario, an extra noise is added to the velocity term to model the uncertainty of the pedestrian’s plan. The model is defined as follows.
  (4a) $$R(v)≜R\left(\left[\begin{array}{c} v_{x}\\ v_{y} \end{array}\right]\right)=\frac{1}{\sqrt{v_{x}^{2}+v_{x}^{2}}}\left[\begin{array}{cc} v_{x} & -v_{y}\\ v_{y} & v_{x} \end{array}\right]$$
  (4b) $$\begin{array}{cc} L_{m^{\prime }} & ≜\left[\begin{array}{cc} \sigma_{vx,m^{\prime }} & 0\\ 0 & \sigma_{vy,m^{\prime }} \end{array}\right]\\ x^{\prime }=g_{m^{\prime },m}\left(x,\epsilon \right)\; & ≜\left[\begin{array}{c} v^{\prime }\Delta t\\ {\bar{v}}_{m^{\prime }}^{\prime }+L_{m^{\prime }}R\left(v\right)\epsilon \end{array}\right], \end{array}$$
  (4c) $$\begin{array}{cc} \;\mathrm{where}\;{\bar{v}}_{m^{\prime }} & =m^{\prime }\cdot \mathrm{SocialForceModel}\left(x\right),\epsilon ∼N\left(0,I\right) \end{array}$$

In the encoding step, we adopt the bimodal extended Kalman filter to estimate the current state distribution of every pedestrian. We take the observed pedestrian position sequence $p^{1:t_{\mathrm{obs}}}$ as the input data, and estimate the state distribution frame by frame recursively. For each frame, the filter takes the observed position and the previous state distribution, and estimates the current state distribution accordingly. Through the steps, the current state distribution is computed, which contains the parameterized variables representing the mode distribution and the base state distribution.

After the current state distribution is estimated, the decoding phase is then applied to the distribution to generate the predicted pedestrian future trajectories. To decode and predict the future trajectory of the pedestrians. First, states are sampled from the computed state distributions. The sampled state is taken as the initial input. The input then goes into the state transition models and predicts the state. Then, the state is generated recursively via the state transition model by taking the previous state as the input, until the required prediction sequence length is reached. To reduce the prediction variance, the velocity noise ϵ is applied to the only states for the output predicted trajectories.

### 2.3. Model Optimization

To obtain the optimal model for predicting the pedestrian trajectories. We have to optimize the proposed model and obtain the optimal parameters. We will illustrate the optimization process for the proposed model.

#### 2.3.1. Observation Model Optimization

For the position observation model $z=h_{m}\left(x,\delta \right)≜p+\sigma_{p}\delta$, it is parameterized via the parameter $\sigma_{p}$ to represent the standard deviation of the observation noise.

To compute such parameter, we first compute the smoothed pedestrian trajectories via the BSpline [24] algorithm, and we denote the smooth trajectory as ${\tilde{p}}_{1:t_{\mathrm{pred}}}$. Now, for every pedestrian position p there is a smoothed version of position $\tilde{p}$. We want to file the parameter $\sigma_{p}$ such that it maximizes the sum of the log likelihood for all positions in the dataset in following equation.

(5) $$\max\prod_{i=1}^{N}N\left(p_{i};{\tilde{p}}_{i},\sigma_{p}^{2}I_{2\times 2}\right)$$

The optimal parameter could be computed by following equation.

(6) $$\sigma_{p}=\sqrt{\frac{1}{2N}\sum_{i=1}^{N}{∥p_{i}-{\tilde{p}}_{i}∥}^{2}}$$

#### 2.3.2. Mode Transition Model Optimization

The mode transition model is a part of the state transition model, and it is used to describe the mode transition probability between the transition of states. The mode transition model is defined as follows.

(7) $$p\left(m^{\prime }|m;T\right)=t_{m^{\prime }|m}$$

We parameterize the state transition as follows:

(8) $$T≜\left[\begin{array}{cc} t_{0|0} & t_{0|1}\\ t_{1|0} & t_{1|1} \end{array}\right]$$

If we let the previous mode distribution be $p\left(m\right)=w_{m}$, the current mode distribution $p\left(m^{\prime }\right)={w^{\prime }}_{m^{\prime }}$, and the predict current mode distribution be $\bar{bel}\left(m^{\prime }\right)={\bar{w}}_{m^{\prime }}$. The process of the prediction could be formulated as follows.

(9) $${\bar{w}}_{m^{\prime },i}^{\prime }=\sum_{m}t_{m^{\prime }|m}w_{m,i}$$

We want to compute the optimal parameter $T^{*}$ such that the sum of square errors between the predicted probability ${\bar{w}}_{m^{\prime },i}^{\prime }$ and the ground-truth $w_{m^{\prime },i}^{\prime }$ is minimized.

(10) $$T^{*}=\underset{T}{\arg\min}\sum_{m}{\left(w_{m^{\prime },i}^{\prime }-{\bar{w}}_{m^{\prime },i}^{\prime }\right)}^{2}$$

Then, we reformulate the equation in the form of matrix multiplication.

(11) $$T^{*}=\underset{T}{\arg\min}\sum_{m^{\prime }}{\left(w_{m^{\prime }}^{\prime }-Wt_{m^{\prime }}\right)}^{T}\left(w_{m^{\prime }}^{\prime }-Wt_{m^{\prime }}\right)$$

where

(12) $$w_{m^{\prime }}^{\prime }=\left[\begin{array}{c} w_{m^{\prime },1}^{\prime }\\ \vdots \\ w_{m^{\prime },N}^{\prime } \end{array}\right],W=\left[\begin{array}{cc} w_{0,1} & w_{1,1}\\ \vdots & \vdots \\ w_{0,N} & w_{1,N} \end{array}\right],t_{m^{\prime }}=\left[\begin{array}{c} t_{m^{\prime }|0}\\ t_{m^{\prime }|1} \end{array}\right]$$

Solve the equation we have the optimal transition as follows.

(13) $$T^{*}={\left[\begin{array}{cc} t_{0} & t_{1} \end{array}\right]}^{T},\;\mathrm{where}\;t_{m^{\prime }}={\left(W^{T}W\right)}^{-1}W^{T}w_{m^{\prime }}^{\prime }$$

#### 2.3.3. Mode Probability Model Optimization

In order to obtain the mode probability $w_{m,i}^{\prime }$ for optimizing the mode transition model, we construct the mode probability model and compute the mode probability base on this model. First, we assume the speed of the pedestrians is distributed as the mixture normal distribution, the distribution is defined as follows:

(14) $$p\left(v;\Theta \right)≜\sum_{m=0}^{1}w_{v,m}N\left(v;\mu_{v,m},\sigma_{v,m}^{2}\right)$$

The model is parameterized with six parameters: two for the mode weight $w_{v,0:1}$, two for the speed mean $\mu_{v,0:1}$, and two for the standard deviation $\sigma_{v,0:1}$, the subscript denotes the mode.

(15) $$\Theta ≜\langle w_{v,0:1},\mu_{v,0:1},\sigma_{v,0:1}\rangle$$

For all the speed data in the pedestrian trajectory dataset, we would like to search for the optimal parameters so that the sum of model log-likelihood could be maximized as follows:

(16) $$\underset{\Theta }{\arg\max}\sum_{i}\log p\left(v_{i};\Theta \right)$$

As we assume the pedestrian prefers to move in either static or moving mode, the velocity noise is different mode is thereby distributed, respectively. We model the velocity noise as normal distribution and distributed, respectively, in each mode. We use the notation $\Theta_{v,m}$ to denote the velocity noise parameter under the specific mode $m^{\prime }$, it is defined as follows:

(17) $$w_{m^{\prime },i}^{\prime }=\frac{w_{v,m^{\prime }}N\left(v_{i}^{\prime };\mu_{v,m^{\prime }},\sigma_{v,m^{\prime }}^{2}\right)}{p(v_{i}^{\prime };\Theta )}$$

#### 2.3.4. Velocity Noise Parameter Optimization

As we assume the pedestrian prefer to move in either static of moving mode. The velocity noise is different mode is therefore distributed, respectively. We model the velocity noise as normal distribution and distributed, respectively, in each mode. We use the notation $\Theta_{v,m}$ to denote the velocity noise parameter under the specific mode $m^{\prime }$, it is defined as follows:

(18) $$\Theta_{v,m^{\prime }}≜\left[\begin{array}{c} \sigma_{vx,m^{\prime }}\\ \sigma_{vy,m^{\prime }} \end{array}\right]$$

From the motion models, we assumes the velocity difference $\Delta v_{m^{\prime }}^{\prime }$ between ground-truth velocity v and the predicted velocity ${\bar{v}}_{m^{\prime }}^{\prime }$ is distributed under a scaled and rotated standard normal distribution:

(19) $$\Delta v_{m^{\prime }}^{\prime }≜v^{\prime }-{\bar{v}}_{m^{\prime }}^{\prime }=RL_{m^{\prime }}\epsilon ,\epsilon ∼N\left(\epsilon ;0,I_{2\times 2}\right),R=\frac{1}{v_{x}^{2}+v_{y}^{2}}\left[\begin{array}{cc} v_{x} & -v_{y}\\ v_{y} & v_{x} \end{array}\right],L_{m^{\prime }}=I_{2\times 2}\Theta_{v,m^{\prime }}$$

We want to maximize the sum of weighted log likelihood as follows:

(20) $$\underset{\Theta_{v,m^{\prime }}}{\arg\max}\sum_{i}w_{m^{\prime },i}^{\prime }\cdot \log N\left(\Delta v_{m^{\prime },i}^{\prime };0,RL_{m^{\prime }}^{2}R^{T}\right)$$

where

(21) $$w_{m^{\prime },i}^{\prime }=\frac{w_{v,m^{\prime }}N\left(v_{i}^{\prime };\mu_{v,m^{\prime }},\sigma_{v,m^{\prime }}^{2}\right)}{p(v_{i}^{\prime };\Theta )}{|}_{v_{i}^{\prime }=∥v_{i}^{\prime }∥}$$

The parameter could be calculated by the following formula, note that the square and square root operators are applied in an element-wise manner.

(22) $$\Theta_{v,m^{\prime }}=\left[\begin{array}{c} \sigma_{vx,m^{\prime }}\\ \sigma_{vy,m^{\prime }} \end{array}\right]=\sqrt{\frac{\sum_{i}w_{m^{\prime },i}^{\prime }{\left(R^{-1}\Delta v_{i}^{\prime }\right)}^{2}}{\sum_{i}w_{m^{\prime },i}^{\prime }}}$$

#### 2.3.5. Social Force Model Optimization

For the pedestrians’ in the moving mode, we model the moving behavior with a motion model:

(23) $${\hat{x}}^{\prime }=\mathrm{SocialForceMotionModel}\left(x;\Theta_{\mathrm{SFM}}\right)$$

We define the model loss to be the average of expected weighted displacement error for pedestrians in the moving mode:

(24) $$L\left(\Theta_{\mathrm{SFM}}\right)=\frac{1}{n}\sum_{i=1}^{n}w_{m^{\prime },i}^{\prime }∥{\hat{p}}_{i}^{\prime }-p_{i}^{\prime }∥{|}_{m=1}$$

We optimize the loss by minimizing the loss via the gradient decent algorithm, the parameters are then eventually retrieved.

## 3. Results

In our experiment, we divide the collected data into two parts. One is the training set for training the models. The other one is the testing set, which is not involved in the training process. The constant velocity model, social force model [11], and social GAN [6] model are selected to be compared with the proposed model (with and without noise). The proposed model is trained and tested in a leave-one-out manner.

As for the configuration details of the testing models. Our model is configured to be tested with both the deterministic and the stochastic prediction settings. The deterministic model uses the same parameters as those of the stochastic model. However, the sampling process is degenerated: the normal distribution is replaced with Dirac delta distribution and the mode is always transit to the most likely one. In the deterministic model the most likely sample is always picked, and the randomness is canceled.

The social force model is configured to adopt the repulsive force terms from other pedestrians and the obstacles but discard the attractive force terms for reaching the destination and some other objects of interest, as those terms are not obvious during the prediction process. Two parameter sets of the social force model [11] are chosen for this experiment. One follows the original parameters settings as shown in [11], and the other follows the parameters that are refined through the training process.

For the social GAN model, the dimension of the hidden state is set to 32 for the encoder and decoder of the generator, and is configured to 64 for the encoder of the discriminator. The input coordinates are embedded as 16-dimensional vectors. The generator and discriminator are iteratively trained with a batch size of 16 using Adam [25] with an initial learning rate of $5\cdot 10^{-4}$ for the generator and $10^{-3}$ for the discriminator. The k-variety loss with k=10 and the pooling mechanism are applied for training this model.

### 3.1. Hardware and Software Environment

In thisstudy, we used Dell XPS 15 laptop as our main computing platform. All calculations and experiments described in the paper were computed with this device, including the collection of the pedestrian point-cloud data, pedestrian trajectory extraction, data curation, and evaluation for all models. The detailed hardware specifications of this computing platform are shown in Table 1.

For the part of the software environment, we used Ubuntu 20.04 as the operating system of our developing environment. We used the C++ programming language and the robotic operating system (ROS) for point-cloud data processing, mapping, and trajectory data curation. We used the Python programming language and the Pytorch machine learning framework for building and testing models. The detailed software specifications are shown in Table 2.

### 3.2. Metrics and Results

We adopt several metrics to help evaluate the complexity and the performance of the proposed model. The metrics include the model parameters count, model prediction run-time, average displacement error, final displacement error, minimum social distance, and minimum physical distance. We will illustrate the definition of these metrics and show the results in the following paragraphs. The metric with the best performance among the compared models is marked with boldface, and the metric with the second-best performance is underlined.

#### 3.2.1. Model Complexity

To evaluate the complexity of the models, we count the number of parameters of the prediction model to show the space complexity of the model. We also measure the average trajectory prediction run-time per scene in the dataset to show the time complexity of the model. The run-time of the model is tested and measured on both the CPU device and GPU device.

Table 3 shows the metrics for all the models to be compared. Note that we only compared the parameters count for the prediction model, but we do not include the auxiliary models such as the discriminator in the social GAN [6] model.

#### 3.2.2. Average Displacement Error

The average displacement error (ADE) is the metric that shows the average displacement between the predicted pedestrian trajectories $\hat{Y}$ and the ground-truth future trajectories *Y*. To compute this metric, the magnitude of the difference between ground-truth position and the prediction position is computed first, then the average is taken for all the frames in a scene. The formula to compute ADE is defined as follows, in which the variable *n* denotes the number of pedestrians in the specified scene.

(25) $$\mathrm{ADE}\left(Y,\hat{Y}\right)≜\frac{1}{n}\cdot \frac{1}{t_{pred}}\sum_{i=1}^{n}\sum_{t=t_{\mathrm{obs}}+1}^{t_{\mathrm{obs}}+t_{\mathrm{pred}}}∥p_{i}^{t}-{\hat{p}}_{i}^{t}∥$$

The average displacement error (ADE) is the metric for single-scene evaluation. To evaluate the performance of the scenes in the dataset. We derive two metrics based on this metric, which are the mean ADE metric and the minimum ADE metric.

The mean ADE metric (meanADE) computes the average ADE for all sampled scenes in the specified dataset. Furthermore, in order to capture the performance of the stochastic models, we sample $N_{\mathrm{samples}}=10$ trajectory predictions for a scene, and compute the average over all the samples as well.

(26) $$\mathrm{meanADE}≜\frac{1}{N_{\mathrm{scenes}}}\sum_{X,Y}\left(\frac{1}{N_{\mathrm{samples}}}\sum_{k}\mathrm{ADE}\left(Y,{\hat{Y}}^{k}\right)\right){|}_{{\hat{Y}}^{k}=G\left(X,O\right)}$$

The minimum ADE metric (minADE) is a metric to capture the average minimum error among the samples. To compute this metric, first, the minimum ADE is evaluated for all prediction samples in each scene, and then the average is taken over the scenes. The computation formula for this metric is defined as follows:

(27) $$\mathrm{minADE}≜\frac{1}{N_{\mathrm{scenes}}}\sum_{X,Y}\left(\min_{k}\mathrm{ADE}\left(Y,{\hat{Y}}^{k}\right)\right){|}_{{\hat{Y}}^{k}=G\left(X,O\right)}$$

The experiment result for the ADE metrics is shown in Table 4.

#### 3.2.3. Final Displacement Error

The final displacement error (FDE) is the metric that shows the average displacement in the last predicted frame between the predicted pedestrian trajectories $\hat{Y}$ and the ground-truth future trajectories *Y*. To compute this metric, the magnitude of the difference between the ground-truth position and the prediction position is computed first, then the average is taken for the last frame in the scene. The formula to compute FDE is defined as follows, in which the variable *n* denotes the number of pedestrians in the specified scene.

(28) $$\mathrm{FDE}\left(Y,\hat{Y}\right)≜\frac{1}{n}\sum_{i=1}^{n}\left(∥p_{i}^{t}-{\hat{p}}_{i}^{t}∥\right){|}_{t=t_{\mathrm{obs}}+t_{\mathrm{pred}}}$$

The final displacement error (FDE) is the metric for single-scene evaluation. To evaluate the performance of the scenes in the dataset. We derive two metrics based on this metric, which are the mean FDE metric and the minimum FDE metric.

The mean FDE metric computes the average FDE for all sampled scenes in the specified dataset. Furthermore, in order to capture the performance of the stochastic models, we sample $N_{\mathrm{samples}}=10$ trajectory predictions for a scene, and compute the average over all the samples as well.

(29) $$\mathrm{meanFDE}≜\frac{1}{N_{\mathrm{scenes}}}\sum_{X,Y}\left(\frac{1}{N_{\mathrm{samples}}}\sum_{k}\mathrm{FDE}\left(Y,{\hat{Y}}^{k}\right)\right){|}_{{\hat{Y}}^{k}=G\left(X,O\right)}$$

The min ADE metric is a metric to capture the average minimum error among the samples. To compute this metric, first the minimum ADE is evaluated for all prediction samples in each scene, and then the average is taken over the scenes. The computation formula for this metric is defined as follows:

(30) $$\mathrm{minFDE}≜\frac{1}{N_{\mathrm{scenes}}}\sum_{X,Y}\left(\frac{1}{N_{\mathrm{samples}}}\sum_{k}\mathrm{FDE}\left(Y,{\hat{Y}}^{k}\right)\right){|}_{{\hat{Y}}^{k}=G\left(X,O\right)}$$

The experiment result for the FDE metrics is shown in Table 5.

#### 3.2.4. Minimum Social Distance

The minimum social distance (MSD) is the metric that computes the minimum distance between the pedestrians among all predicted frames. The metric helps us grasp the idea of how the model handles and avoids social collisions.

(31) $$\mathrm{MSD}\left(\hat{Y}\right)≜\min_{t,i,j\neq i}∥{\hat{p}}_{i}^{t}-{\hat{p}}_{j}^{t}∥$$

To further apply the metric for the scenes in the dataset. We derive three metrics based on this metric, these three metrics are the min MSD metric, the 5th% MSD metric, and the Social collision ratio (SCR) metric. To compute these metrics, the MSD metric for each scene in the dataset is computed first. Then, the minimum MSD metrics among all the MSD metrics are found and defined as the minimum MSD metric. The 5th% MSD metric is computed by taking the 5th% value of the percentile of the MSD metrics in the dataset. As for the Social collision ratio, it is defined as the ratio of collision between pedestrians in the dataset, we consider a scene has collision if the MSD metric for the scene is less than 20 cm.

The experiment result for the MSD metrics is shown in Table 6.

#### 3.2.5. Minimum Physical Distance

The minimum physical distance (MPD) is the metric that computes the minimum distance between the pedestrians among all predicted frames. This metrics help us the grasp the idea how the model handle and avoid the physical collisions.

(32) $$\mathrm{MPD}\left(\hat{Y},O\right)≜\min_{t,i,k}∥{\hat{p}}_{i}^{t}-o_{k}∥$$

To further apply this metric to the scenes in the dataset. We derive three metrics based on this metric, these three metrics are the min MPD metric, the 5th% MPD metric, and the physical collision ratio (PCR) metric. To compute these metrics, the MPD metric for each scene in the dataset is computed first. Then, the minimum MPD metrics among all the MPD metrics are found and defined as the minimum MPD metric. The 5th% MPD metric is computed by taking the 5th% value of the percentile of the MPD metrics in the dataset. As for the physical collision ratio, it is defined as the ratio of collision between the pedestrian and the physical obstacles in the dataset, we consider a scene has a collision if the MPD metric for the scene is less than 20 cm. The experiment result for the MPD metrics is shown in Table 7.

### 3.3. Graphical Results

**Figure 4:**
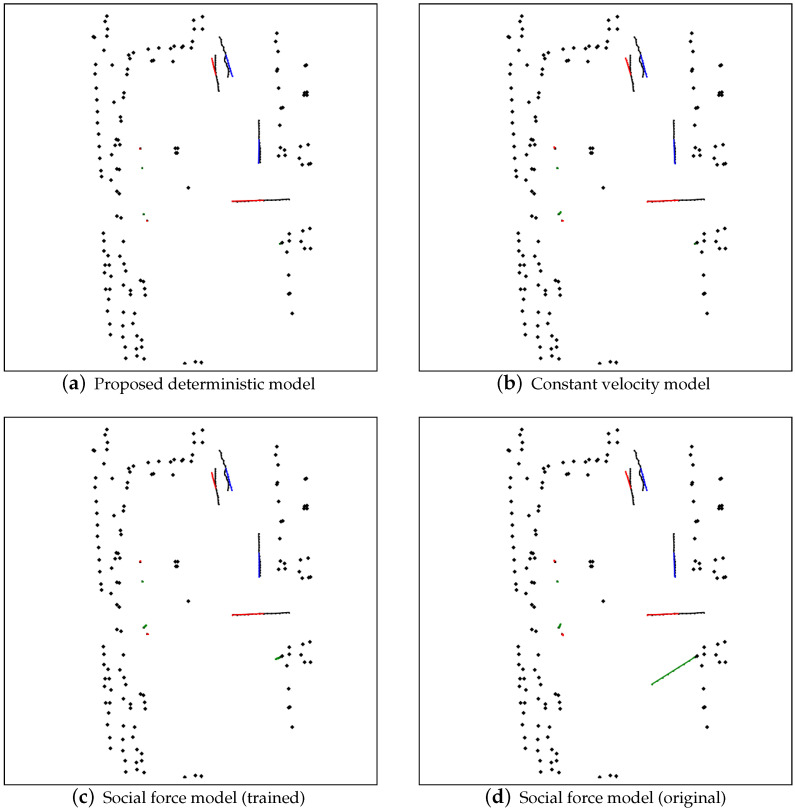
Graphical result of the compared deterministic models.

Figure 4 shows the predicted trajectories and comparison results with the ground-truth trajectories for deterministic models, including our proposed model, constant velocity model and the social force model [11] in two configurations. The predicted trajectories of the pedestrians in this scene are lines colored either red, green, or blue for every individual. The ground-truth trajectories are represented with black lines. The black dots mark the locations of the physical obstacles in the experiment environment. Each figure shows the 60 by 60 square meters area and is represented in the resolution of 600 × 600 pixels.

**Figure 5:**
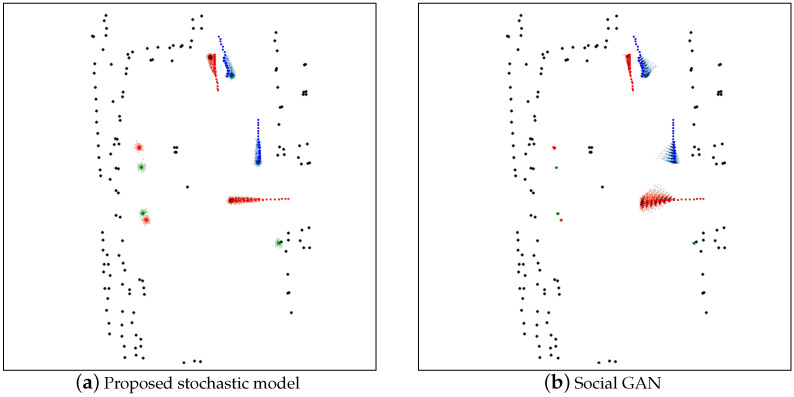
Graphical result of the compared stochastic models.

Figure 5 shows the predicted trajectories distribution compared with the ground-truth trajectories for two stochastic models. The trajectories of the pedestrians in this scene are colored either red, green, or blue for every individual. The predicted trajectories distribution is generated by stacking 1000 predictions into the 2D histogram and is represented using the heatmap. The ground-truth trajectories are represented with solid dots. The black dots mark the locations of the physical obstacles in the experiment environment. Each figure shows the 60 by 60 square meters area and is represented in the resolution of 600 × 600 pixels.

These figures demonstrate that the proposed model can predict in a bimodal manner, i.e., the pedestrian prefers to maintain his/her position in static mode while moving with the consideration of the environment in the moving mode. When compared with other deterministic models such as constant velocity and the social force model, we see those models often suffers drifts for the pedestrian which is supposed to standing still at the same spot, as shown by those 4 pedestrians in the left and the pedestrian at the right-bottom by an obstacle in Figure 4. Furthermore, the ground-truth trajectories fall in the predicted distribution for both our proposed stochastic model and the social GAN model [6], which showcase the prediction coverage of the models.

## 4. Discussion

From the experimental result in the previous section, we could see that our traditional model-based model uses slightly more parameters than the social force model [11], as we introduce the bimodal extended Kalman filtering mechanism in addition to the social force model. Nevertheless, our model uses much fewer parameters than that of the deep learning-based model such as the social GAN [6] model.

The run-time of the proposed models is longer than that of all the other compared models, which is mainly due to the following three reasons. First, our model has an encoding step, which processes the observed trajectories and estimates the states of the pedestrians. This cause our model appears slower than that of the constant velocity model and social force model [11]. Second, our model considers and computes the social and physical interaction on a frame-by-frame basis. When compared with the social GAN [6], which only computes the social interaction feature once per trajectory. Thus, our frame-by-frame approach requires extras computations more than those that do not. Finally, our model requires the computation of Jacobian matrices for approximation during the encoding phase, which is also a time-consuming operation compared with normal feedforward operations.

For the performance of the ADE and FDE metrics. Our proposed deterministic model outperforms all compared models on the mean ADE metric and the mean FDE metric. Our proposed models also outperform all compared models except for the social GAN [6] model, when comparing the minimum ADE metric and the minimum FDE metric. By considering the bimodal moving nature of the pedestrian with the proposed bimodal extended Kalman filter, our proposed model shows the improvement in performance when compared with the constant velocity model and the social force model as they do not utilize such property. Regarding the metrics compared with the social GAN [6] model, such a result is reasonable, as social GAN [6] is optimized on its variety loss. Optimizing the variety loss will encourage the variety of the predicted trajectories while keeping the minimum displacement error as small as possible. However, such loss might also cause the mean ADE to be larger due to the diversity of the prediction.

As for the performance of the MSD and MPD metrics, our proposed models and the social force model [11] perform better than that by the constant velocity model and the social GAN model for all the MSD and MPD metrics while remaining competitive with that by the social force model. The proposed models have larger distances in terms of MSD and MPD metrics and lower collision ratio values. These metrics show that our model does gain the social avoidance ability thanks to the social force model [11] being applied in the moving mode, while the original social force model performs the best in these metrics, it is reasonable as the model is configured to have a larger repulsive force for both the social and physical obstacles.

## 5. Conclusions

We build a trajectory dataset from the point-cloud data collected by the 3D LiDAR sensor in a semi-automatic labeling manner and proposed a bimodal extended Kalman filter to model the bimodal moving nature of the pedestrian. We then adopt the social force model [11] as the motion model for the pedestrian in moving mode, and the constant position model for the pedestrian in the static mode. With this approach, the proposed model shows increased performance in ADE and FDE metrics than of those models which are not aware of the bimodal motion properties. Furthermore, our model inherits the ability from the social force model to avoid collisions with pedestrians and physical obstacles. In addition, our proposed approach is fully explainable as comparing with those deep learning-based model, which justifies the usefulness of the proposed approach.

## Figures and Tables

**Table 1:** Hardware specification.

Hardware	Specification
Computer	Dell XPS 15 9570
RAM	8ATF1G64HZ-2G6E1 SODIMM DDR4 Micron 8GiB * 2
CPU	Intel Core i7-8750H CPU @ 2.20 GHz; 6 core; 12 threads
GPU	GeForce GTX 1050 Ti with Max-Q Design/PCIe/SSE2
Storage	THNSN5512GPUK NVMe TOSHIBA 512GB
LiDAR	Velodyne VLP-16

**Table 2:** Software environment.

Software	Version
OS	Ubuntu 20.04.1 LTS
ROS	Noetic Ninjemys
C++	14
GCC	9.4.0
Python	3.8.10
PyTorch	1.12.1+cu113
NVIDIA Driver	470.74
CUDA	11.4

**Table 3:** Model Complexity.

	Number of Parameters	Run-Time (CPU)	Run-Time (GPU)
Constant Velocity	**0**	**36.2** µs	**73.7** µs
Social GAN	60,234	3.3 ms	3.0 ms
SFM (trained)	7	9.0 ms	15.1 ms
SFM (original)	7	9.9 ms	15.3 ms
Proposed (deterministic)	14	66.3 ms	78.1 ms
Proposed (stochastic)	14	68.5 ms	78.1 ms

**Table 4:** Evaluation of ADE metrics.

	Training Set		Testing Set	
	meanADE	minADE	meanADE	minADE
Ground Truth	0 ± 0	0 ± 0	0 ± 0	0 ± 0
Constant Velocity	0.262 ± 0.088	0.262 ± 0.088	0.260 ± 0.086	0.260 ± 0.086
Social GAN	0.231 ± 0.071	**0.196 ± 0.062**	0.251 ± 0.079	**0.215 ± 0.070**
SFM (trained)	0.270 ± 0.085	0.270 ± 0.085	0.268 ± 0.083	0.268 ± 0.083
SFM (original)	0.468 ± 0.395	0.468 ± 0.395	0.469 ± 0.273	0.469 ± 0.273
Proposed (deterministic)	**0.218 ± 0.087**	0.218 ± 0.087	**0.215 ± 0.084**	0.215 ± 0.084
Proposed (stochastic)	0.331 ± 0.120	0.243 ± 0.084	0.329 ± 0.114	0.241 ± 0.079

**Table 5:** Evaluation of FDE metrics.

	Training Set		Testing Set	
	meanFDE	minFDE	meanFDE	minFDE
Ground Truth	0 ± 0	0 ± 0	0 ± 0	0 ± 0
Constant Velocity	0.480 ± 0.169	0.480 ± 0.169	0.476 ± 0.165	0.476 ± 0.165
Social GAN	0.412 ± 0.138	**0.342 ± 0.119**	0.453 ± 0.154	**0.381 ± 0.137**
SFM (trained)	0.504 ± 0.164	0.504 ± 0.164	0.500 ± 0.160	0.500 ± 0.160
SFM (original)	0.887 ± 1.427	0.887 ± 1.427	0.887 ± 0.823	0.887 ± 0.823
Proposed (deterministic)	**0.402 ± 0.172**	0.402 ± 0.172	**0.395 ± 0.164**	0.395 ± 0.164
Proposed (stochastic)	0.594 ± 0.240	0.407 ± 0.164	0.590 ± 0.226	0.403 ± 0.155

**Table 6:** Evaluation of MSD Metrics.

	Training Set			Testing Set		
	minMSD	5th%MSD	SCR	minMSD	5th%MSD	SCR
Ground Truth	0.204	0.266	0%	0.227	0.286	0%
Constant Velocity	0.006	0.110	12.3%	0.014	0.115	12.7%
Social GAN	0.003	0.176	6.4%	0.006	0.176	6.4%
SFM (trained)	0.086	0.350	0.7%	0.127	0.351	0.7%
SFM (original)	**0.106**	**0.583**	**0.2%**	**0.207**	**0.588**	**0.2%**
Proposed (deterministic)	0.033	0.312	0.9%	0.085	0.328	1.0%
Proposed (stochastic)	0.012	0.209	4.5%	0.021	0.211	4.6%

**Table 7:** Evaluation of MPD metrics.

	Training Set			Testing Set		
	minMPD	5th%MPD	PCR	minMPD	5th%MPD	PCR
Ground Truth	0.319	0.472	0.0%	0.408	0.674	0.0%
Constant Velocity	0.023	0.382	1.4%	0.072	0.429	1.3%
Social GAN	0.014	0.405	0.9%	0.016	0.442	1.2%
SFM (trained)	0.073	0.491	0.3%	**0.219**	0.679	0.2%
SFM (original)	**0.102**	**0.623**	0.2%	0.188	0.619	0.2%
Proposed (deterministic)	0.072	0.477	**0.2%**	0.167	**0.697**	**0.2%**
Proposed (stochastic)	0.011	0.401	0.8%	0.030	0.527	0.8%

## Data Availability

The data presented in this study are openly available at https://github.com/cylin-at-research/ntut_library (last accessed on 23 October 2022).

## Abbreviations

The following abbreviations are used in this manuscript:

LSTM	Long Short-Term Memory
GAN	Adversarial Generative Network
SFM	Social Force Model
MOT	Multi-Object Tracking
LiDAR	Light Detection Furthermore, Ranging
ROS	Robotic Operating System
ADE	Average Displacement Error
FDE	Final Displacement Error
MSD	Minimum Social Distance
MPD	Minimum Physical Distance
SCR	Social Collision Ratio
PCR	Physical Collision Ratio
